# Interrelationship of Stress, Environment, and Herpes Simplex Virus Type-1 on Behçet’s Disease: Using a Mouse Model

**DOI:** 10.3389/fimmu.2021.607768

**Published:** 2021-03-31

**Authors:** S. M. Shamsul Islam, Hye-Myung Ryu, Hasan M. Sayeed, Seonghyang Sohn

**Affiliations:** ^1^ Department of Biomedical Science, School of Medicine, Ajou University, Suwon, South Korea; ^2^ Department of Microbiology, Ajou University School of Medicine, Suwon, South Korea

**Keywords:** Behçet’s disease, stress, environment, herpes simplex virus, gut microbiota, mouse model

## Abstract

The purpose of this study was to investigate effects of stress and environment factors on the induction of Behçet’s disease (BD) using HSV-1 infected mouse model. BD is a chronic multisystemic inflammatory disease of unknown etiology. Environmental factors, immune dysfunction, and herpes simplex virus type-1 (HSV) infection might be triggers of BD. To investigate effects of environmental factors on the incidence of BD, HSV was inoculated into mice. Mice were then maintained in conventional facility or SPF facility to compare BD incidence rates. The incidence of BD was also tracked by adding stressors such as substance P (anxiety stress), 4°C (cold stress), xanthine sodium salt (oxidative stress), or 77 dB noise (noise stress). To clarify immune mechanisms involved in the difference in BD incidence caused by various stresses, dendritic cell activation markers were analyzed using flow cytometry. The combination of conventional environment, noise stress, and HSV had the highest rate of BD (38.1%) among all groups. However, HSV inoculated group in a SPF environment had the lowest incidence (2.2%). Frequencies of dendritic cell activation markers such as CD40, CD83, CD80, and CD86 were expressed differently under various stresses. Noise stress increased frequencies of CD83 positive cells. Noise stress also upregulated transcription factors T-bet and ROR-γt. Different gut microbiota compositions were observed between SPF and conventional environment by 16S rRNA sequence analysis. Environment and stress influenced the incidence of HSV-induced BD. Microbial diversity due to environmental differences might be one explanation for regional differences in the incidence of BD.

## Introduction

Behçet’s disease (BD) is a chronic, recurrent, multisystemic variable vessel vasculitis inflammatory disorder that can cause oral and genital ulcers, papulopustular and nodular skin lesions, arthritis, uveitis, venous and arterial thrombosis, arterial aneurysms, central nervous system lesions, and gastrointestinal ulcers (1). Several studies have addressed the epidemiological complex of BD. However, the exact etiology of BD remains inconclusive. Several factors are involved in the development of BD, including environmental pollutants, viral or bacterial infections, genetic polymorphism, and immune dysregulations (2). Herpes Simplex Virus (HSV) has been isolated from patients’ ocular fluids (3), saliva (4), skin lesions (5), and peripheral blood (6). It is considered a triggering factor of BD. HSV-1 inoculated ICR mice had BD-like symptoms such as oral ulcers, genital ulcers, skin ulcers, eye symptoms, intestinal ulcers, arthritis, and skin crust (7). Therefore, it has been used as a potential animal model for BD.

Stresses such as psychological, environmental, and physiological stressors are known to threaten bodies’ homeostasis and nonspecific responses (8). Stress has diverse adverse health effects, many of which are mediated by stressful actions on immune system (9). It has been reported that stress can induce pro-inflammatory or type 1-type 2 cytokine-mediated immune responses, leading to inability to modulate innate and adaptive immune responses (9). Interaction between physical and psychological factors and low quality of life are associated with BD progression (10). Stress is also recognized as a modulator of gut microbiota (11). Gut microbiota is a key component of immune homeostasis (12). Dysbiosis, an imbalance of gut microbiota, has been observed in many inflammatory autoimmune diseases such as multiple sclerosis (13), rheumatoid arthritis (14), and inflammatory bowel disease (15). It has been also found that BD is associated with significant gut microbiota changes, suggesting alterations in gut microbiota might play a role in the pathogenesis of BD (16). During psychological stress, the migration of dendritic cells (DCs) is enhanced, resulting in increased T cell responses (17). DCs are known as the forefront initiator of immune signals and the most potent antigen presenting cells with the ability to activate and differentiate T lymphocytes. They can effectively connect innate and adaptive immune systems. Antigen-capturing DCs go through a maturation process. Mature DCs can differentiate naïve T cells, B cells, and NK cells (18). DCs are involved in several autoimmune diseases such as inflammatory bowel disease (IBD) (19), rheumatoid arthritis (RA) (20), uveitis (21), and Crohn’s disease (CD) (22). During the maturation process of DCs, DCs accumulate peptides, major histocompatibility complex class II molecules, and costimulatory molecules. CD40, CD80, CD83, and CD86 are DC costimulatory molecules (23). Among them, CD83 is known to play a pathogenic role in BD mice (24). It has been reported that inhibition of CD83 by GC7 can affect the ability of DCs to induce T cell proliferation (25).

In the present study, an HSV-1 induced BD symptomatic mice model was used to investigate the mechanism by which stress could increase the incidence of BD. DCs and gut microbiota compositions were also studied to understand their involvement in BD. Findings of this study revealed that DC and gut microbiota were regulated under stress conditions that caused BD.

## Materials and Methods

### Animal Experiment

Animals used in this study were handled according to animal protocols and guidelines established by the Ethics Review Board of Ajou University School of Medicine. All animal experiments were approved by the Institutional Animal Care and Use Committee (IACUC) of Ajou University (IACUC-2018-0017). Institute of Cancer Research (ICR) mice at 4 to 5 weeks old were used as previously described (7). Virus inoculation was performed and then observed for 16 weeks. Mice were bred in a specific pathogen free (SPF) room or a conventional room with temperature and light control (20-22°C, 12 h light/dark cycle). These mice had free access to food and water. Animals were closely monitored and photographed during experiments.

### HSV-1 Induced BD Symptomatic Mice

Virus inoculation was performed using published procedures (7). Briefly, the earlobes of mice were scratched with a needle and inoculated with 20 μL of 1 x 10^6^ pfu/mL of HSV-1 (F strain) grown in Vero cells. Virus inoculation was performed twice with an interval of 10 days. Mice were anesthetized by intraperitoneal injection with 2,2,2-tribromo ethanol (240 mg/kg), virus was inoculated, and BD symptoms were confirmed by follow-up for 5 to 16 weeks. Several symptoms, including oral ulcers, genital ulcers, erythema, skin pustules, skin ulcers, arthritis, diarrhea, red-eye, loss of balance, and facial edema, were observed in mice after HSV inoculation. The incidence of BD was between 2% and 38% in HSV inoculated mice. Oral, genital, skin ulcers, and eye symptoms were classified as major symptoms. Arthritis, intestinal ulceration, and neurological involvement were considered as minor symptoms. Mice with at least one major symptom and at least one minor symptom were classified as having BD. Each symptom score was one point. The sum of scores of different symptoms was used to determine the severity of BD using 2006 BD Current Activity Form prepared by the International Society for Behçet’s Disease (http://medhealth.leeds.ac.uk/download/910/behcetsdiseaseactivityform). All BD mice used in this study showed ocular symptoms. The disappearance of symptoms or a reduction in skin lesion size of 20% or more indicated improvement of BD. Whether ocular symptoms were improved was not confirmed. HSV-1 inoculated and asymptomatic mice were used as BD normal (BDN) mice as previously described (7).

### Induction of Stress

Mice were exposed to several types of stress. For noise stress induction, mice were exposed to noise in a noise chamber. The ventilation chamber was equipped with a high-frequency driver (Electro-Voice DH-7) and a power amplifier (Ax5505, Inkel) operated with an arbitrary waveform generator (Agilent 33210A) that generated sound waves in the range of 0.1 Hz to 15 MHz. Animals were exposed to a 77 dB sound pressure level of broadband noise measured with a dual channel real-time frequency analyzer (Type 2144, Bruel & Kjaer) for 1 hour daily for 10 consecutive days. 77 dB sound pressure is a typical construction site noise level. Each animal was placed in a separate 10 cm diameter wire mesh cage to eliminate sound pressure attenuation by adjacent animals. For the induction of anxiety stress, Substance P (SP) was injected intraperitoneally. For cold stress induction, mice were kept in a cold room at 4°C for one hour daily for 10 consecutive days. And for induction of oxidative stress, xanthine sodium salt was injected intraperitoneally.

### Preparation of siRNA

CD83 siRNA preparation was performed as detailed previously (24). In brief, CD83 siRNA oligonucleotides with the following sense and antisense sequences were synthesized by Integrated DNA Technology (Coralville, IA, USA): Sense, 5’-GUGCUUUUCAGUCAUCUACAAGCTA-3’; Antisense, 3’-CUCACGAAAAGUCAGUAGAUGUUCGAU-5’.

### Mouse Treatment

SP (5 µg) was injected intraperitoneally five times every other day into normal mice, HSV-1 induced single symptomatic mice, and BD mice. To induce oxidative stress, 10 mg Xanthine sodium salt was injected intraperitoneally into mice once daily for five consecutive days. As a control group, mice were treated with PBS for the same period. CD83 siRNA was prepared as previously described (24). CD83 siRNA was mixed with a transfection reagent JetPEI (Polyplus-transfection, Illkirch-Graffenstaden, France) for injection into mice. To apply siRNA to mice, 0.5 µmol CD83 siRNA was dissolved in 200 µL of 5% glucose solution, mixed with jetPEI, and injected intraperitoneally twice at 5-day intervals. As a control, mice were treated with only transfection reagent during the same period.

### Flow Cytometric Analysis

Mice were euthanized and peripheral blood leukocytes (PBLs) were collected by cardiac puncture and peritoneal macrophages (pMQs) were isolated from the peritoneal cavity. PBLs were washed with ammonium-chloride-potassium (ACK) lysis buffer to lyse red blood cells. pMQs and PBLs were washed with phosphate buffered saline (PBS). Then 1x10^6^ cells were stained with Percp-eFluor-labeled anti-mouse CD40, eFluor 660-labeled anti-mouse CD83, PE-Cyanine7-labeled anti-mouse CD80, and FITC-labeled anti-mouse CD86 (eBioscience, San Diego, CA, USA) at 4°C in the dark for 30 min. Stained cells were analyzed with FACS Aria III flow cytometer (Becton Dickinson, San Jose, CA, USA) with x10,000 gated cells.

### Analysis of Microbiota in Mouse Feces by 16S Ribosomal RNA (rRNA) Gene Sequencing

Fresh feces were collected from mice to analyze mouse gut microbiota based on 16S rRNA gene amplicon sequence. 16S rRNA gene sequencing of V3 and V4 amplicons was performed using 16S rRNA gene PCR primer (Forward Primer 5′-TCG TCG GCA GCG TCA GAT GTG TAT AAG AGA CAG CCT ACG GGN GGC WGC AG-3′, Reverse Primer 5′-GTC TCG TGG GCT CGG AGA TGT GTA TAA GAG ACA GGA CTA CHV GGG TAT CTA ATC C-3′). The number of operational taxonomic units (OTUs) was determined by clustering the sequence from each sample with 97% sequence identities as a cut-off using quantitative insights into microbial ecology (QIIME) software (v.1.8.0). Taxonomic abundance was counted with a National Center for Biotechnology Information (NCBI) database using a confidence threshold of 0.8 derived from preprocessed reads for each sample.

### Total RNA Extraction and Quantitative RT-PCR

Total RNAs were isolated from cells using TRIzol reagent (Thermo Fisher, Waltham, MA, USA) according to the manufacturer’s recommendations. RNAs were quantified with a NanoDrop spectrophotometer (NanoDrop Technologies, Wilmington, NC, USA) and reverse-transcribed into cDNA using Prime Script cDNA Synthesis kit (Takara Shuzo Co., Otsu, Shiga, Japan). qRT-PCR was performed on a 7500 Real-time PCR system (Applied Biosystems) in duplicate for each target transcript using SYBER green PCR Master Mix (Applied Biosystems, Foster City, CA, USA) and gene-specific primers. For qRT-PCR, 1 µL cDNA served as template. The final reaction volume was 20 µL. Each reaction contained gene-specific primers listed in **(** **Table 1** **)**. The PCR condition was as follows: 2 min at 94°C, followed by 40 cycles at 94°C for 3s, 55°C for 30s, and 72°C for 30s, and a final extension step at 72°C for 10 min. Relative gene expression level was calculated using the 2^-ΔΔCt^ method and expression level of each gene was normalized against the level of β-actin, a house-keeping gene. Data are presented as fold changes relative to untreated control.

**Table 1:** List of primers used in real-time PCR.

**Gene Name**	**Primer Sequence (Forward)**	**Primer Sequence (Reverse)**
**Egr2**	5′-CTCCCGTATCCGAGTAGC-3′	5′-GATGCCCGCACTCACAAT-3′
**Ripk2**	5′- ATCCCGTACCACAAGCTCG-3′	5′-GGATGTGTAGGTGCTTCACTG-3′
**Ptpn1**	5′-GGCTATTTACCAGGACATTCGAC-3′	5′- TCCGACTGTGGTCAAAAGGG-3′
**Cebpb**	5′- CAACCTGGAGACGCAGCACAAG-3′	5′-GCTTGAACAAGTTCCGCAGGGT-3′
**T-bet**	5′-ATGTTTGTGGATGTGGTCTTGGT-3′	5′-CGGTTCCCTGGCATGCT-3′
**ROR-γt**	5′-GACCCACACCTCACAAATTGA-3′	5′-AGTAGGCCACATTACACTGCT-3′
**Gata-3**	5′-CAAGCTTCATAATACCCCTGACTATG-3′	5′-GCGCGTCATGCACCTTTT-3′
**Foxp3**	5′-CACAATATGCGACCCCCTTTC-3′	5′-AACATGCGAGTAAACCAATGGTA -3′
**β-actin**	5′- TGTCCACCTTCCAGCAGATGT-3′	5′-AGCTCAGTA ACAGTCCGCCTAG-3′

### Statistical Analysis

Statistical differences between experimental groups were determined using Graphpad Prism (version 8.3.1) for Windows (Graphpad Software, La Jolla, CA, USA) and MedCalc^®^ version 9.3.0.0. (MedCalc, Ostend, Belgium). Statistical significance was assessed by performing Mann-Whiteny U test and Student’s *t*-test. Statistical significance was considered when *p-*value was less than 0.05.

## Results

### Effects of Stress and Environment on the Incidence of BD in Mice

To determine the effect of environment on the prevalence of BD, HSV was inoculated into mice and the prevalence of BD was compared between mice in SPF environment and a conventional facility. Under conventional conditions, HSV inoculated mice showed higher incidence of BD than those under SPF conditions (15.0% *vs.* 2.2%, *p* = 0.03). To investigate the effect of stress on the prevalence of BD, HSV inoculated mice were given several types of stress. Types of stress applied to mice included anxiety, cold, oxidative, and noise stress. SP was used to induce anxiety stress. In order to induce cold stress, mice were placed in a cold chamber at 4°C. To induce noise stress, mice were placed inside a 77-dB noise-producing device. HSV inoculated and SP treated mice under a conventional condition showed higher incidence of BD than HSV inoculated mice in an SPF condition (17.10% vs. 2.2%, *p* = 0.04). Mice inoculated with HSV and exposed to noise stress under conventional conditions had significantly higher incidence rate of BD (38.1% vs. 2.2%, *p* = 0.0001) than HSV inoculated mice under SPF conditions. Noise stress additionally treated to HSV-inoculated mice under the conventional conditions increased the incidence of BD compared to HSV inoculated mice (38.10% *vs.* 15.00%, *p* = 0.04) **(** **Table 2** **)**. However, oxidative stress and cold stress failed to increase the rate of BD incidence.

**Table 2:** Differences in incidence of Behçet’s disease between mice maintained in a conventional facility and those maintained in a specific pathogen free (SPF) facility.

Mouse Environments	Application	BD/Total Number of Mice (%)
SPF	HSV	1/45 (2.2%)
Conventional	HSV	6/40 (15.0%)*
	HSV + Substance P (Anxiety stress)	7/41 (17.1%)*
	HSV + Noise stress	8/21 (38.1%)**#

#p < 0.05 vs. HSV (Conventional).

*p < 0.05 vs. HSV (SPF).

**p < 0.001 vs. HSV (SPF).

### Altered Gut Microbial Compositions in Normal Mice Under Conventional and SPF Conditions

**Figure 1:**
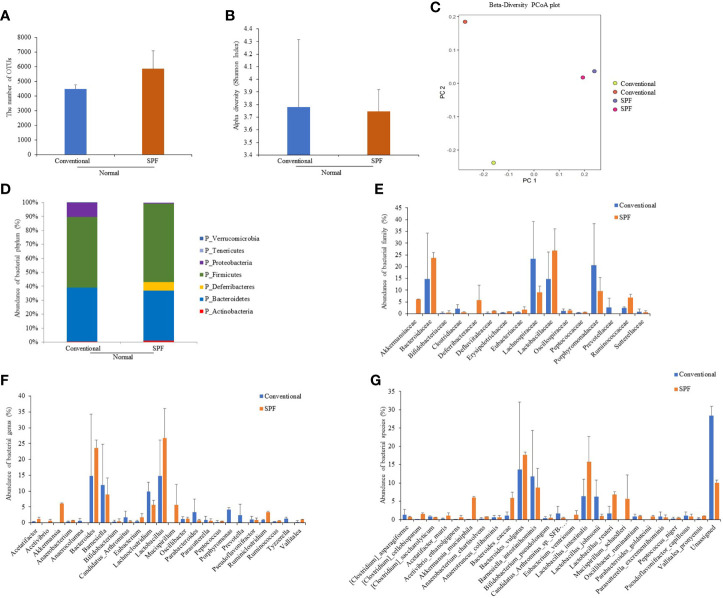
Fecal samples were collected from normal mice maintained under conventional or SPF condition and 16S rRNA amplicons were sequenced for analysis. Microbiome analysis was performed using sequencing data of 16S rRNA V3 and V4 amplicons. The number of OTUs **(A)** and Alpha diversity (Shannon index) **(B)**, and Principal component analysis (PCA) plot **(C)** were calculated. Phylum **(D)**, family **(E)**, genus **(F)**, and species **(G)** showed significant differences in fecal 16S rRNA sequence analysis between groups. Two mice were used in each group.

To determine differences in compositions of gut microbiota according to the environment in which mice were maintained, microbiota were analyzed for fresh feces samples of mice (five weeks old). Microbiome was determined based on 16S rRNA sequence analysis. Based on a NCBI classifier comparison of microbial community, taxonomy construct distances between conventional and SPF conditions were determined using Shannon diversity index and beta diversity and visualized by Principal Coordinate Analysis (PCoA) plot (**Figures 1A–C**). Based on fecal sample microbiome analysis, the number of OTUs was increased in SPF-maintained mice compared to that in conventionally maintained mice. There was no significant difference in alpha diversity between groups (**Figures 1A, B**). For mice maintained in the conventional facility, *Firmicutes*, *Bacteroids*, and *Proteobacteria* were the most prevalent phyla, while *Firmicutes*, *Bacteroids*, and *Deferribacteres* were the most prevalent phyla in mice maintained in the SPF facility (**Figure 1D**). *Akkermansiaceae*, *Defluviitaleaceae, Erysipelotrichaceae*, and *Ruminococcaceae* were microbial families that were more dominant in mice under the SPF condition than in mice under the conventional condition. *Lachnospiraceae*, *Porphyromonadaceae*, and *Prevotellaceae* were more dominant microbial families in mice maintained in the conventional condition than in mice maintained in the SPF condition (**Figure 1E**). At genus level, *Akkermansia, Lactobacillus, Mucispirillum, Ruminiclostridium, Ruminococcus, and Vallitalea* groups had higher populations in mice maintained under the SPF condition than mice maintained in the conventional condition. At genus level, *Candidatus, Lachnoclostridium, Parabacteroidetes, Porphyromonas, Prevotela*, and *Tyzzerella* had higher populations in mice under the conventional condition than in mice under the SPF condition, although such differences were not statistically significant (**Figure 1F**). For bacterial species, *Clostridium cellobioparum*, *Akkermansia muciniphila*, *Anaerobacterium chartisolvens*, *Bacteroides caccae*, *Lactobacillus intestinalis*, *Lactobacillus reuteri*, *Mucispirillum schaedleri*, and *Parabacteroides goldsteinii* were more dominant in mice maintained under the SPF condition, whereas *Candidatus arthromitus* and *Lactobacillus johnsonii* were more dominant in mice maintained under the conventional condition, although differences between the two groups were not statistically significant (**Figure 1G**). Differences in fecal microbiota between conventional and SPF conditions and *p*-values are shown in **Supplementary Table ST1**.

#### Frequencies of Dendritic Cell Activation Markers CD40, CD83, CD80, and CD86-Expressing Cells in BD Mice

**Figure 2:**
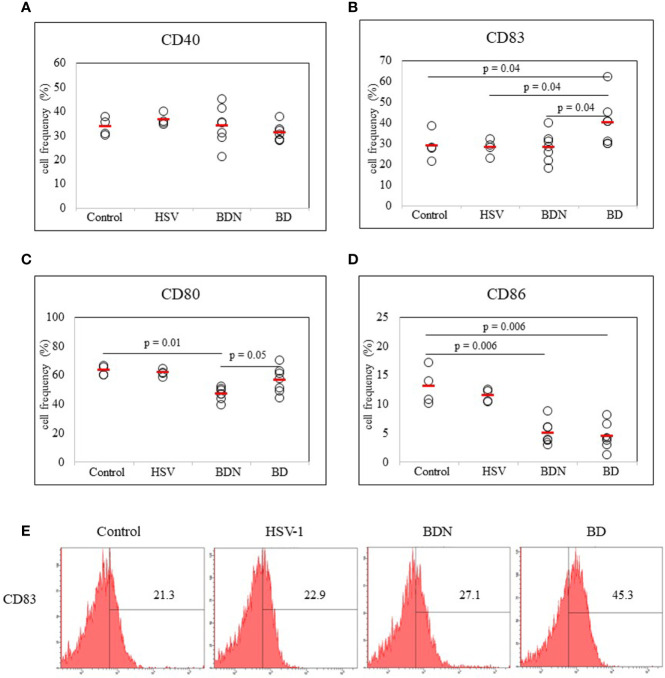
Frequencies of DC costimulatory molecules CD40, CD83, CD80, and CD86 in peripheral blood leukocytes (PBLs) were evaluated in normal, HSV-1 inoculated, BDN, and BD mice. Isolated PBLs were analyzed by flow cytometry after surface staining **(A–D)**. Representative histogram of CD83+ cells is shown in **(E)**. For statistical analysis, the Mann-Whitney U test was performed with GraphPad. Experiments were performed independently at least three times.

To determine whether there was a correlation between DC activation marker expression and BD mice with ocular symptoms, frequencies of cells expressing DC activation markers were analyzed in mice PBLs. Frequencies of activated DC molecules CD40+, CD83+, CD80+, and CD86+ cells in normal, HSV-1 inoculated, BDN, and BD mice were determined by flow cytometric analysis (FACS). Frequencies of CD83+ cells were significantly increased in BD symptomatic mice (40.00 ± 11.51%) than in BDN (28.12 ± 7.14%, *p* = 0.04), HSV inoculated (28.17 ± 3.92%, *p* = 0.04), and normal mice (28.87 ± 7.12%, *p* = 0.04) (**Figure 2B**). Frequencies of CD80+ cells were significantly higher in BD mice than in BDN mice (56.50 ± 8.98% *vs.* 46.94 ± 4.28%, *p* = 0.05) (**Figure 2C**). Frequencies of CD86+ cells were reduced in BD (4.42 ± 2.6%, *p* = 0.006) and BDN (4.98 ± 2.07%, *p* = 0.006) mice than in normal mice (12.97 ± 3.20%) (**Figure 2D**). There were no significant differences in frequencies of CD40+ cells among groups (**Figure 2A**). A representative histogram of CD83 is shown in (**Figure 2E**)

#### Identification of Several Genes Expressed in Eye Symptomatic BD Mice by Real-Time PCR

**Figure 3:**
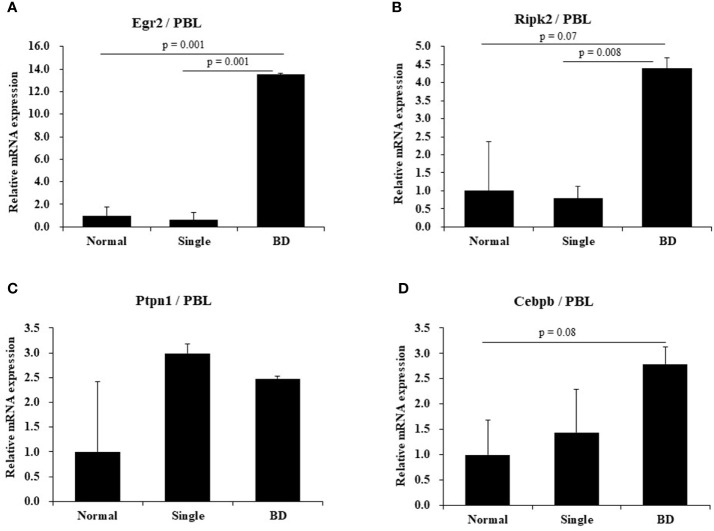
Expression levels of several mRNAs in BD mice by real-time PCR. Expression levels of several mRNAs in peripheral blood leukocytes (PBLs) of BD mice with ocular symptoms were analyzed by real time-PCR **(A–D)**. Selected genes were Egr2, Ripk2, Rtpn1, and Cebpb. After normalization against β-actin, relative expression levels were analyzed for each sample. The experiment was conducted in duplicate.

To determine expression levels of several genes expressed in BD mice with ocular symptoms, real-time PCR was performed using mouse PBLs. Results revealed that mRNA expression levels of Egr2 were higher in BD mice with eye symptoms than in mice with eye symptoms only (13.50 ± 0.14 *vs.* 0.6 ± 0.70, *p* = 0.001) and in normal mice (13.50 ± 0.14 *vs.* 1.0 ± 0.73, *p* = 0.001) (**Figure 3A**). Ripk2 mRNA expression levels were also higher in BD mice with eye symptoms than in mice with eye symptoms only (4.39 ± 0.30 *vs.* 0.78 ± 0.35, *p* = 0.008) and in normal mice (4.39 ± 0.30 *vs.* 1.0 ± 1.36, *p* = 0.07) (**Figure 3B**). Cebpb mRNA expression levels in PBLs of BD mice with ocular symptoms were also higher than in normal mice, although the difference between the two groups was not statistically significant (2.79 ± 0.34 *vs.* 1.00 ± 0.69, *p* = 0.08) (**Figure 3D**). Ptpn1 mRNA expression levels did not differ significantly among the groups (**Figure 3C**). The mice used in this experiment were 2 for each group.

#### Identification of Several Stressors That Influence the Activation of Dendritic Cells

**Figure 4:**
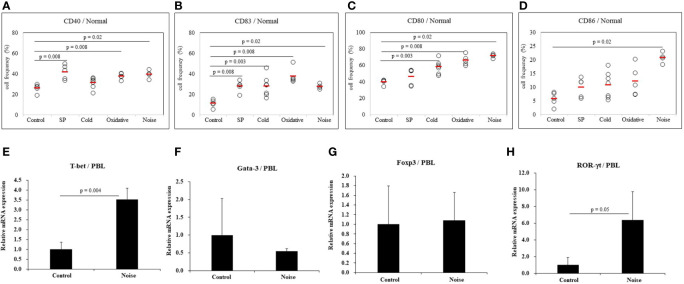
Expression levels of dendritic cell activation markers in normal mice after stress treatment. Frequencies of DC activation molecules CD40+, CD83+, CD80+, and CD86+ cells in normal mice after stress treatment were analyzed by FACS analysis **(A–D)**. Substance P (SP) was used for anxiety stress, 4°C temperature was used for cold stress, xanthine sodium salt was used for oxidative stress, and 77dB sound was used for noise stress induction. For statistical analysis, Mann-Whitney U test was performed. Cellular transcription factor mRNA expression levels in PBLs of normal mice exposed to noise stress were analyzed by real time-PCR **(E–H)**.

To determine whether stress might affect the activation of dendritic cells, several types of stress were used to treat normal mice. Frequencies of cells expressing DC activation markers were measured by FACS. DC markers analyzed in PBLs of mice were CD40, CD83, CD80, and CD86. To induce anxiety stress, substance P was administered to mice by intraperitoneal injection. Frequencies of CD40 positive (+) and CD83+ cells were significantly increased in mice under anxiety stress than in control mice (41.80 ± 7.19% *vs.* 25.94 ± 4.16%, *p* = 0.008; 27.78 ± 5.45% *vs.* 11.20 ± 3.88%, *p* = 0.008, respectively) (**Figures 4A, B**). In mice treated with cold stress, frequencies of CD83+ and CD80+ cells were significantly increased than in control mice (27.94 ± 9.90% *vs.* 11.20 ± 3.88%, *p* = 0.003; 58.10 ± 8.05% *vs.* 39.90 ± 3.13%, *p* = 0.003, respectively) (**Figures 4B, C**), no significant differences were observed for the frequencies of CD40+ and CD86+ cells (**Figures 4A, D**). In mice treated with xanthine sodium salt for oxidative stress, frequencies of CD40+, CD83+, and CD80+ cells were significantly increased than in control mice (37.68 ± 3.04% *vs.* 25.94 ± 4.16%, *p* = 0.008; 37.68 ± 7.63% *vs.* 11.20 ± 3.88%, *p* = 0.008; 66.16 ± 6.11% *vs.* 39.90 ± 3.13%, *p* = 0.008, respectively) (**Figures 4A–C**). In mice treated with noise stress, frequencies of CD40+, CD83+, CD80+, and CD86+ cells were significantly increased than in control mice (39.53 ± 4.06% *vs.* 25.94 ± 4.16%, *p* = 0.02; 27.60 ± 2.40% *vs.* 11.20 ± 3.88%, *p* = 0.02; 71.57 ± 2.67% *vs.* 39.90 ± 3.13%, *p* = 0.02; 20.72 ± 2.00% *vs.* 5.70 ± 2.46%, *p* = 0.02, respectively) (**Figures 4A–D**). Noise stress treatment also regulated transcription factors in PBLs of normal mice. T-bet and ROR-γt mRNA expression levels were increased in mice treated with noise stress than in control mice (3.5 ± 0.6 *vs.* 1.0 ± 0.4, *p* = 0.004; 6.4 ± 3.4 *vs.* 1.0 ± 0.9, *p* = 0.05, respectively) (**Figures 4E, H**). There were no significant differences in Gata-3 or Foxp3 mRNA expression between the two groups (**Figures 4F, G**). The mice used in (**Figures 4E–H**) were 3 for each group. Results of each stress dose are shown in supplementary Figures (**Supplementary Figures S1**–**S3**).

#### Stress Activates Dendritic Cells in HSV-Infected State

**Figure 5:**
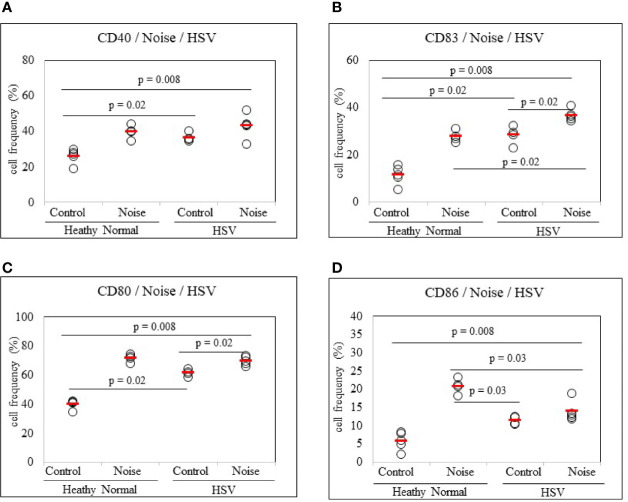
Frequency analysis of DC-stimulating molecules CD40, CD83, CD80 and CD86-expressing cells in normal mice and HSV-1 inoculated mice with or without noise stress. Mice were exposed to noise stress for 10 days after the first HSV-1 inoculation. One day after second inoculation of HSV-1, peripheral blood leukocytes were applied to flow cytometry **(A–D)**. Experiments were conducted independently at least three times.

To determine whether stress could promote dendritic cell activation in HSV-infected state, noise stress was applied to HSV inoculated mice and DC activation markers in PBLs were analyzed by FACS. Noise stress increased frequencies of CD80+ and CD83+ cells in HSV inoculated mice than in control mice (69.68 ± 3.02% *vs.* 61.42 ± 2.30%, *p* = 0.02; 36.62 ± 2.41% *vs.* 28.17 ± 3.92%, *p* = 0.02, respectively) (**Figures 5B, C**). HSV infection itself also increased frequencies of CD40+, CD83+, and CD80+ cells than in healthy, uninfected mice (36.40 ± 2.52% *vs.* 25.94 ± 4.16%, *p* = 0.02; 28.17 ± 3.90% *vs.* 11.20 ± 3.88%, *p* = 0.02; 61.42 ± 2.30% *vs.* 39.90 ± 3.13%, *p* = 0.02, respectively) (**Figures 5A–C**). On the other hand, HSV infection itself or with noise stress down-regulated frequencies of CD86+ cells than in uninfected noise stress induced mice (11.42 ± 1.12% *vs.* 20.72 ± 2.00%, *p* = 0.03; 13.82 ± 2.78% *vs.* 20.72 ± 2.00%, *p* = 0.03, respectively) (**Figure 5D**).

#### Stress Modulates Dendritic Cell Activation in BD Mice

**Figure 6:**
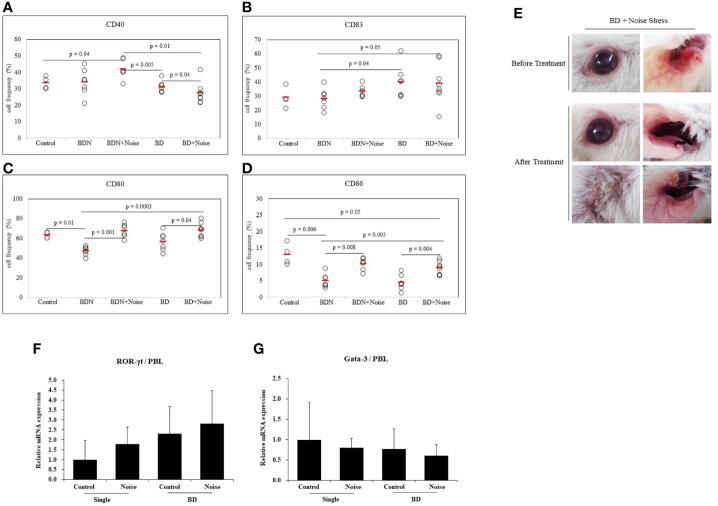
Noise stress affects DC activation in BD mice. To investigate the correlation between noise stress and DC activation in BD, frequencies of DC activation markers CD40, CD83, CD80, and CD83 positive cells in peripheral blood leukocytes of noise-treated BD mice and BDN mouse were measured by flow cytometry **(A–D)**. BD mice under noise stress showed deteriorated or new symptoms **(E)**. Experiments were conducted independently at least three times. mRNA expression levels of cell transcription factors ROR-γt and Gata-3 in mice with only ocular symptoms and BD mice with ocular symptoms were analyzed by real time PCR. Both groups of mice were compared with mice in noise stress treated groups **(F, G)**. Statistical analysis was performed with Student’s *t*-test. The experiment was conducted in duplicate.

To determine whether stress could affect additional activation of dendritic cells, noise stress was applied to BD mice. As a control group, asymptomatic mice (BD Normal, BDN) after HSV administration were used. Results revealed that noise stress decreased frequencies of CD40+ cells in BD mice (31.00 ± 3.50% *vs.* 27.30 ± 6.39%, *p* = 0.04) (**Figure 6A**). Noise stress did not significantly affect frequencies of CD83+ cells in BD mice (**Figure 6B**), although it increased frequencies of CD80+ cells in BD mice (56.50 ± 8.98% *vs.* 68.36 ± 7.23%, *p* = 0.04) and BDN mice (46.94 ± 4.28% *vs.* 67.51 ± 6.99%, *p* = 0.001) (**Figure 6C**). Noise stress also increased frequencies of CD86+ cells in BD mice (4.42 ± 2.26% *vs.* 8.86 ± 2.00%, p = 0.004) and BDN mice (4.98 ± 2.07% *vs.* 10.10 ± 1.94%, *p* = 0.008) (**Figure 6D**). BD symptomatic mice exposed to noise stress had worse symptoms or developed new symptoms than non-stressed BD mice (**Figure 6E**). In single-symptomatic mice (before BD stage) and in BD mice, noise stress increased ROR-γt mRNA expression levels but decreased GATA-3 mRNA levels, although such increase or decrease was not statistically significant (**Figures 6F, G**). The mice used in **Figures 6F, G** were 3 for each group.

#### Inhibition of CD83 Ameliorates BD Symptoms Even After Exposure to Noise Stress

**Figure 7:**
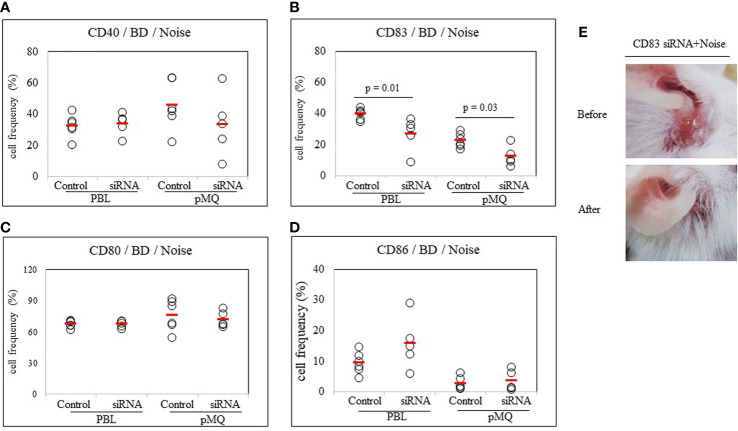
CD83 siRNA reduces frequencies of CD83+ cells in BD mice. Both noise stress and CD83 inhibition were used to treat BD mice at the same time. Symptoms were improved and frequencies of CD83+ cells were decreased in the group that showed inhibition of CD83 after treatment with CD83 siRNA. BD mice were treated with noise stress and CD83 inhibition at the same time and changes in symptoms and frequencies of DC activation markers were analyzed **(A–D)**. In the group that showed inhibition of CD83 after treatment with CD83 siRNA, frequencies of CD83+ cells were decreased **(B)**. CD83 siRNA inhibited the increase in frequencies of CD83+ cells induced by noise stress and inhibited the deterioration of BD symptoms **(E)**. For statistical analysis, Mann-Whitney U test was performed. Experiments were conducted independently at least three times.

To confirm the role of CD83 in stress-treated BD mice, noise stress and CD83 inhibition were simultaneously used to treat BD mice and changes in symptoms were tracked. CD83 inhibition was performed with CD83 siRNA. Frequencies of CD83+ cells in PBLs and peritoneal macrophages were then analyzed by FACS. BD mice exposed to noise stress were treated with CD83 siRNA and compared to BD mice exposed to noise stress only. Frequencies of CD83+ cells were significantly downregulated in mice treated with both CD83 siRNA and noise stress than in mice treated with noise stress only (39.38 ± 3.53% *vs.* 26.66 ± 10.96%, *p* = 0.01; 22.42 ± 4.55% *vs.* 12.18 ± 6.42%, *p* = 0.03, respectively) (**Figure 7B**). Treatment of BD mice exposed to noise stress with CD83 siRNA improved BD symptoms (**Figure 7E**). There were no differences of CD40+, CD80+, or CD86+ cells with or without CD83 siRNA treatment in noise stress-treated BD mice (**Figures 7A, C, D**).

### Discussion

The incidence of BD varies worldwide, ranging from 3.3 to 31.8 cases per 100,000 population (26). Even in countries around the Silk Road, the prevalence of BD varies in the Middle East, Far East, and Europe. Currently, microbiota research is actively underway. Many researchers expect that microbiota can explain differences in the incidence of BD under various environmental conditions of different regions. Dysbiosis of the gut microbiome may trigger or exacerbate the inflammatory process in BD, and a reduced intestinal microbial diversity was observed in patients with BD (27). In an animal study, fecal microbiota transplantation using feces from BD patients has been shown to significantly exacerbate uveitis (16). Gut microbiota has been reported to differ between laboratory-controlled animals and animals maintained in outdoor facilities (28). To determine the relationship between microbiota and BD outbreak, BD prevalence experiments were conducted in mice maintained in different environmental facilities. We demonstrated the higher incidence of BD in mice maintained in conventional facilities than in mice maintained in SPF facilities, suggesting normal microflora can influence BD induction. Mice maintained in the SPF facility displayed a greater number of OTUs and more bacterial phyla than mice maintained in the conventional facility. This suggests bacterial phylum diversity is associated with inhibition of BD development. Mice maintained in SPF facilities showed a greater number of *Akkermansia muciniphilia*, *Bacteroides caccae*, *Lactobacillus reuteri*, and *Parabacteroides goldsteinii* than in mice maintained in conventional facilities. *Akkermansia muciniphilia* is known to reduce IFN-γ production (29). *Bacteroides caccae* decreased IL-8 levels (30). IL-8 was upregulated in patients with active BD (31). *Lactobacillus reuteri* increased butyrate production, which alleviated airway inflammation (32), and *Parabacteroides goldsteinii* has been suggested for use as a novel probiotic in obesity and type 2 diabetes (33). This suggests environment plays a crucial role in alteration of gut microbiota.

It has been reported stress-induced vascular disease and inflammatory symptoms are associated with microbiota changes (34, 35). Stress has a variety of adverse health effects, some of which are mediated by its action on the immune system (9). In the present study, various stresses were applied to HSV-1 inoculated mice for induction of BD to determine what stresses might play an important role in BD induction. Of various stresses, noise stress was the most effective in increasing the incidence of BD in mice. Mice are sound sensitive animals (36). Although noise stress has a significant effect on the induction of BD in mice, there is still no evidence as to whether it could have the same effect on BD development in human patients. Therefore, more research is needed to determine types of stress that can affect the prevalence of BD in humans. In BD patients, stress has been defined as the most common self-reporting trigger for oral ulcer development (37).

Repeated social defeat stress can activate DC and enhance cytokine secretion (38). DC plays an important role in a variety of human and murine autoimmune diseases (39). Mature DCs express co-stimulatory molecules CD40, CD80, CD83, and CD86 on cell surfaces. These molecules can regulate inflammation (40, 41). Among DC co-stimulatory molecules, CD83 is one of the markers of DC maturation or activation. It carries out functional interactions between DCs and lymphocytes (42). An increase in CD83 expressing DC has been observed in patients with Crohn’s disease (43) and in patients with rheumatoid arthritis (44). In the present study, frequencies of CD83 expressing cells were significantly increased in BD mice, suggesting CD83 plays an important role in BD inflammation.

Stress can suppress immune functions in some conditions, while provoking immune functions in other conditions (45). Acute stress increases blood levels of proinflammatory cytokines (46). Stressful life events are also provoking factors of BD (47). Stress can increase DC maturation by enhancing activation markers. Increased DC trafficking from skin to lymph nodes under stress has been observed (45). In uveitis, DC promotes the activation of different subsets of Th cells (48). Excessive Th1/Th17 responses in ocular BD patients can be suppressed by modulating DC function through immunomodulatory therapy (49). Oxidative stress enhances DC maturation and increases frequencies of CD86+ and CD40+ cells in mouse bone marrow-derived DC cultures (50). Substance P, a marker of pain and anxiety stress, increases DC maturation in mice skin by regulating MHCII, ICAM-1, CD11c, and langerin (51). However, there have been no reports about regulation of CD40, CD80, CD83, and CD86 by substance P. Temperature stress can also influence CD86+ DC frequencies in laboratory mice (52).

In Chinese Han BD patients, uveitis is correlated with expression levels of EGR2, RIPK2, CEBPB, LACC1, and PTPN1 (53). We found that BD mice with ocular symptoms showed increased expression levels of Egr2, Ripk2, and Cebpb mRNAs than normal mice. BD mice with ocular symptoms had similar expression patterns to those with BD patients with uveitis.

Elevated levels of Th1 and Th17 cytokines may play an important role in the pathogenesis of BD (54). Expression levels of T-bet (T-box expression in T cells) mRNA supporting the Th1 subset of T cells are increased in BD patients with uveitis (55). ROR-γt mRNA levels and frequencies of IL-17A+ Th17 cells are elevated in BD patients with uveitis (56). Results of the present mouse study shows noise stress increased T-bet and RORγt mRNA expression levels. Stress-induced Th1 and Th17 immune responses might contribute to BD inflammation response.

Proinflammatory cytokines promoted DC maturation (57). Inhibition of CD83 could significantly prevent DC-mediated T cell activation (25, 58). In our previous reports, inhibition of CD83, with CD83 siRNA, was able to improve BD symptoms in mice (24). In the current study, noise stress treated BD mice improved when CD83 was suppressed. This means that CD83 expression may be a more potent modulator than the stress in BD. This suggests CD83 is a potential molecule that can control BD symptoms.

In conclusion, we observed environmental conditions and noise stress may play important roles in BD and that it may exacerbate BD symptoms by modulating DC activating markers. Among the DC activating markers, CD83 is a powerful molecule that regulates BD symptoms. The composition of the gut microbiota was regulated according to environmental conditions. Stress management and gut microbiota control may be complementary approaches to BD management.

## Data Availability Statement

The 16S rRNA metagenomic data has been deposited under NCBI accession number PRJNA664645.

## Ethics Statement

The animal study was reviewed and approved by Institutional Animal Care and Use Committee (IACUC) of Ajou University (IACUC-2018-0017).

## Author Contributions

All authors contributed to the article and approved the submitted version. SS contributed to the concept and design of this study, data analysis, and data interpretation. SI, H-MR, and HS contributed to data acquisition, data analysis, and data interpretation. SS and SI wrote the manuscript.

## Funding

This research was supported by a grant (2017R1D1A1B03032168) from the Basic Science Research Project through the National Research Foundation (NRF) funded by the Ministry of Education, Science, and Technology, Republic of Korea. It was also supported by grant (2020R1A2C2012721) through the NRF funded by the Ministry of Science and ICT, Republic of Korea.

## Conflict of Interest

The authors declare that the research was conducted in the absence of any commercial or financial relationships that could be construed as a potential conflict of interest.

## Supplementary Material

Supplementary Figure 1 Frequencies of DC co-stimulatory molecules CD83, CD80, and CD86 in peripheral blood leukocytes (PBLs) of normal mice were elevated after noise stress induction. Isolated PBLs were analyzed by flow cytometric after surface staining **(A–D)**. For statistical analysis, one-way analysis of variance (ANOVA) was performed using GraphPad. Experiments were conducted independently at least three times.

Supplementary Figure 2 Substance P (SP) for anxiety stress increased frequencies of DC co-stimulatory molecules CD40 and CD83 in peripheral blood leukocytes (PBLs) of normal mice. Isolated PBLs were analyzed by flow cytometry after surface staining **(A–D)**. For statistical analysis, one-way ANOVA was performed using GraphPad. Experiments were conducted independently at least three times.

Supplementary Figure 3 Frequencies of DC co-stimulatory molecules CD40, CD83, and CD80 in peripheral blood leukocytes (PBLs) of normal mice are elevated after inducing oxidation using xanthine sodium salt. Isolated PBLs were analyzed by flow cytometry after surface staining **(A–D)**. For statistical analysis, one-way ANOVA was performed using GraphPad. Experiments were conducted independently at least three times.
